# An Airflow-Based Thermal–Tactile–Olfactory Display: Performance Evaluation Under AC and DC Airflow Conditions

**DOI:** 10.3390/s26165139

**Published:** 2026-08-14

**Authors:** Rıza Ilhan

**Affiliations:** Mechanical Engineering Department, Istanbul Aydin University, Besyol, 34295 Istanbul, Turkey; gilkhanisarkandi@aydin.edu.tr

**Keywords:** haptics, multimodal tactile display, airflow, thermal feedback, olfaction

## Abstract

**Highlights:**

**What are the main findings?**

- A multimodal airflow-based tactile–olfactory display was developed and successfully delivered perceivable thermal and odor stimuli with approximately 90% recognition accuracy.
- Airflow rate and modulation frequency significantly affected thermal perception thresholds, with higher flow rates enhancing heat transfer, and higher frequencies increasing warm thresholds while reducing cold thresholds.

**What are the implications of the main findings?**

- Airflow can serve as a unified medium for delivering tactile, thermal, and olfactory feedback, simplifying the design of multimodal haptic interfaces.
- The results provide design guidelines for controlling thermal sensations through airflow dynamics, supporting the development of more immersive VR/AR, telepresence, and human–machine interaction systems.

**Abstract:**

Developing a multimodal tactile display is a key area of interest for haptic scientists, with researchers continuously exploring new methods to achieve this goal. This study introduces a tactile–olfactory display capable of providing touch, temperature, and odor feedback. The display utilizes airflow to deliver feedback to the user, incorporating two air sources and thermoelectric components (Peltier elements). Unlike traditional technologies, it employs convection-based temperature stimulation, where air passing over the thermoelectric modules cools or warms, resulting in temperature modulation. The system was tested under steady airflow conditions (DC airflow) and frequency-modulated airflow conditions (AC airflow). First, a finite element simulation was conducted in Ansys to gain insights into the system parameters. This was followed by experimental evaluations to extract its characteristics and assess its performance. The results indicate that not only the type of airflow but also its rate and frequency play significant roles in rendering surface parameters. Additionally, this airflow-based tactile display has potential applications in both contact and noncontact haptic technologies.

## 1. Introduction

Haptic technology allows users to touch, feel, and interact with virtual objects through tactile feedback. In addition to visual and auditory experiences, haptics establish a physical connection with virtual objects, ensuring better sensory interaction. In this way, users feel as if they truly exist in the virtual world, making their interactions more meaningful and natural [1].

Tactile perception is the sum of sensory information that people experience while interacting with their environment. Tactile perception consists of various components, primarily cutaneous and kinesthetic stimuli. Cutaneous stimuli include tactile sensations such as pressure and vibration felt on the skin [2,3,4,5], while kinesthetic stimuli involve movement and position information perceived through muscles and joints in the body [6,7,8]. The harmonious functioning of these components enables users to accurately perceive sensory information when in contact with a surface or object. Effectively modeling perceptual components in the design of haptic systems is crucial for creating (modeling) more realistic and immersive user experiences [9,10,11].

In recent years, haptic displays have been continuously evolving to enhance the user experience and make human–machine interaction more realistic and efficient. By overcoming the limitations of traditional displays that cater only to visual and auditory senses, these technologies integrate different sensory features such as touch, temperature, and even smell, enabling multisensory interaction with virtual objects [12,13]. Research into haptic displays in application areas such as education, rehabilitation, and virtual reality (VR) demonstrates that haptic technology has the potential to enhance human–machine interaction, making information acquisition and development processes more effective and efficient [14,15,16,17].

### 1.1. Touch Feedback

Surface texture directly shapes users’ perception and is a crucial parameter in the ergonomic design, aesthetics, and functionality of products [18,19,20,21]. Electrovibration and ultrasonic vibration technologies are widely used to simulate surface texture and are particularly effective in tactile feedback systems. Electrovibration generates vibrations at different frequencies using an AC electrical signal, allowing users to perceive the roughness of surfaces in virtual environments [22,23,24,25]. Ultrasonic vibrations, on the other hand, use high-frequency piezoelectric-driven waves to simulate surfaces more precisely and are commonly preferred in medical and robotic applications [26,27]. These technologies not only enhance surface texture perception but also make interactions in digital environments more realistic and immersive. However, their implementation and control require complex engineering solutions.

### 1.2. Thermal Feedback

Thermal feedback is a crucial component of tactile realism, as temperature changes strongly influence material perception and emotional valence [28,29]. Studies in psychophysics and physiology have demonstrated that cold and warm sensations arise from distinct thermoreceptor populations with different response dynamics [30,31]. Warm perception depends mainly on sustained increases in skin temperature, whereas cold perception is driven by rapid decreases and transient cooling rates [32]. Accordingly, temporal patterns, such as the rate of change and modulation frequency of a thermal stimulus, can significantly alter the perceived intensity and comfort of thermal sensations [33,34]. For example, Mekjavic et al. [33] showed that dynamic cooling and heating phases yield asymmetric comfort thresholds, while Wang et al. [30] demonstrated that warm signaling involves inhibitory interactions with cold-sensitive fibers. Chu [35] further reported that fluctuating airflow patterns modify perceived thermal comfort even when mean air temperature remains constant. These findings suggest that the temporal and convective properties of airflow play a decisive role in thermal perception.

Haptic technologies simulate temperature perception, reducing the gap between virtual and physical experiences [28]. Thermoelectric systems and smart materials dynamically adjust surface temperature, allowing users to feel hot or cold objects in virtual environments. This advancement strengthens sensory feedback in fields such as gaming, education, and medicine, bringing virtual experiences even closer to physical reality [29,32]. Recent advances in thermal haptic interfaces have also focused on compact heating technologies for virtual reality applications. Bakha et al. [36] introduced a microheater-based thermal feedback system capable of generating localized heating sensations in immersive virtual environments, demonstrating the feasibility of MEMS-based thermal actuators for VR applications. Building upon this concept, the authors later developed a MEMS-based microheater integrated with a virtual reality system for medical applications, achieving improved thermal rendering performance while emphasizing device miniaturization and precise temperature control [37]. Although these systems provide localized thermal feedback with compact hardware, they are primarily limited to thermal stimulation. In contrast, the proposed system combines thermal, tactile, and olfactory feedback using airflow as a common transmission medium, enabling multimodal interaction while also systematically investigating the effects of airflow rate and frequency modulation on human thermal perception.

### 1.3. Sense of Smell or Olfaction

Smell is one of the most powerful human senses, offering a more holistic and immersive experience when combined with other senses. Integrating odor into haptic displays enhances realism, particularly in virtual reality (VR) and education-related applications. Olfactory devices are used to emit odors from virtual objects, creating a more advanced user experience. Integrating olfactory cues into virtual environments has emerged as a powerful mechanism for enhancing immersion, presence, and cross-modal learning. The broader efficacy of these multisensory systems is documented by Garcia-Ruiz et al. [38], who emphasize the growing role of olfactory displays in optimizing pedagogical outcomes within immersive education and vocational training. To successfully achieve this sensory immersion, research has tackled both spatial delivery algorithms and diverse hardware ergonomics. Wang et al. [39] presented a comprehensive review of olfactory displays, highlighting the fundamental principles, recent technological developments, current applications, and future research challenges in achieving multisensory immersion. Their review emphasizes that integrating olfactory feedback with visual, auditory, and haptic stimuli can substantially improve users’ sense of presence and realism in virtual environments. In addition to stationary olfactory displays, wearable odor delivery systems have attracted considerable attention due to their portability and suitability for mobile and immersive applications. Dobbelstein et al. [40] developed InScent, a wearable olfactory display designed to deliver odor-based notifications, demonstrating that olfactory cues can effectively convey information in mobile interaction scenarios. Similarly, Huang and Chen [41] investigated the use of olfactory cues as interface notifications on mobile devices and reported that odor-based notifications can successfully communicate contextual information while enriching the user experience. These studies demonstrate the growing interest in incorporating olfactory feedback into interactive systems. However, most existing research has focused primarily on odor delivery or notification mechanisms rather than the integration of synchronized thermal, tactile, and olfactory feedback within a unified multimodal interface.

### 1.4. Airflow Stimulation

As the speed of airflow increases, greater pressure is felt on the skin. When airflow comes into contact with human skin, it is detected by specialized cells called mechanoreceptors. These cells sense changes in pressure, speed, and temperature applied to the skin and transmit this information to the brain [27,42,43,44]. Relatively little research has examined how airflow parameters such as flow rate and temporal modulation (frequency) influence thermal and tactile perception at the skin. In a study by Shultz et al. [45], a noncontact feedback system based on an air jet was introduced. Using a speaker, air pulses were sent to create a stimulus. A well-known noncontact application is a mid-air tactile surface that utilizes ultrasonic air jets to create tactile feedback [46,47]. In another technology developed by Arai et al. [48], a micro-venturi array of nozzles was used to create feedback on the fingertip. The micro-fabricated device was demonstrated to have the capability for shape rendering. Building upon these insights, our preliminary study [49] demonstrated the feasibility of employing airflow as a common medium for tactile and olfactory feedback in a multimodal interface.

Previous works on airflow-based tactile systems have primarily focused on force or shape rendering [46,48], whereas the integration of airflow-mediated thermal and odor feedback has remained largely unexplored. More recently, Campos da Silveira et al. [50] developed TWIRL, a temperature-controlled airflow system designed to enhance immersive media experiences by integrating thermal feedback with airflow generation. Similarly, Matsukura et al. [51] introduced the Smelling Screen, an olfactory display capable of presenting virtual odor sources through controlled airflow, while Nakamoto et al. [52] further combined wearable olfactory displays with computational fluid dynamics to improve odor delivery in virtual environments. Although these studies demonstrate the feasibility of airflow-based multisensory interaction, they provide limited quantitative investigation of the influence of airflow dynamics on human thermal perception.

Several studies have investigated airflow as a medium for thermal feedback without incorporating olfactory stimulation. The FIRE system demonstrated mid-air thermo-tactile rendering for immersive virtual environments through localized airflow and thermal stimulation [53]. He et al. [54] proposed a thermal mid-air tactile interface for stress regulation using affective stroking, while Nakajima et al. [55] generated cooling sensations remotely through ultrasound-driven airflow. These studies primarily focused on hardware implementation and application-specific demonstrations rather than systematically characterizing the perceptual effects of airflow parameters.

Airflow has also been employed exclusively for tactile feedback. Lee introduced the Wind Tactor, a wearable airflow-based tactile display capable of rendering directional wind sensations [56]. Hamazaki et al. [57] later proposed ALCool, which combined airflow with evaporative cooling to generate localized cold sensations for ubiquitous haptic applications. Although these systems successfully demonstrated airflow-induced tactile sensations, they did not investigate multimodal integration with thermal and olfactory feedback.

In contrast, the present study introduces a multimodal airflow-based thermal–tactile–olfactory display that provides touch, temperature, and odor feedback using controlled air modulation. Both steady (DC) and frequency-modulated (AC) airflows were investigated through simulation and user experiments to examine how airflow rate and modulation frequency affect human perception of warmness and coldness. The findings contribute to a deeper understanding of unsteady convective heat transfer in haptic interfaces and provide design guidelines for future multimodal display technologies.

## 2. Methodology

### 2.1. Theoretical Simulation

A computational fluid dynamics (CFD) analysis was conducted using ANSYS Fluent (2023 R2, ANSYS Inc., Canonsburg, PA, USA) to investigate the airflow and temperature distribution within the system. The model was designed to provide a detailed understanding of the airflow path from the inlet to the outlet and the heat transfer process occurring along this path. The three-dimensional geometry included a conical air inlet, an aluminum heat sink, and a perforated outlet channel. The model was prepared in ANSYS SpaceClaim (2023 R2, ANSYS Inc., Canonsburg, PA, USA) and refined to ensure compatibility with the simulation environment.

During the meshing process, the grid density was increased near the walls and around the heat sink fins to accurately resolve the steep gradients of velocity and temperature. A mesh independence study confirmed that further refinement caused less than a 2% variation in temperature predictions, indicating that the current mesh resolution was sufficient for reliable results.

Considering the turbulent nature of the flow, the Realizable k–ε turbulence model was selected due to its robustness in high-Reynolds-number internal flows and its stable performance in regions with flow separation and recirculation. The Enhanced Wall Treatment method was applied to capture near-wall effects accurately, maintaining y^+^ values between 1 and 30, which allowed for precise resolution of the viscous sublayer and improved prediction of heat and momentum transfer near solid boundaries.

**Figure 1:**
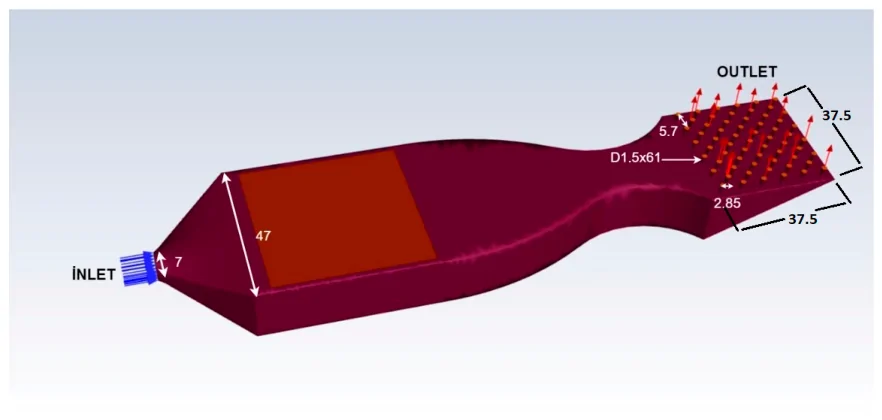
All system parameters were tested in Ansys. Air enters the system through a 7 mm diameter circular cross-section. Later, it is passed between two Peltier modules that either warm up or cool down. The modulated air is guided through the channel toward the tactile display, including holes (all units in mm).

A pressure-based steady-state solver was employed, and the energy equation was activated to account for thermal interactions. Air was modeled as an ideal gas, while the heat sink material was defined as aluminum with a thermal conductivity of 205 W/m·K. The air entered the system through a 7 mm diameter opening (see Figure 1), where a velocity inlet boundary condition was defined, corresponding to flow rates ranging from 10 to 19 L/min. At the outlet, a pressure outlet boundary condition was imposed, allowing the fluid to exit freely at ambient pressure. Most wall surfaces were considered adiabatic, while selected regions included a defined convective heat transfer coefficient (h) to represent wall–air thermal interaction. The thermoelectric module (Peltier element) was modeled as a fixed temperature boundary condition, varying from –10 °C to +90 °C.

**Figure 2:**
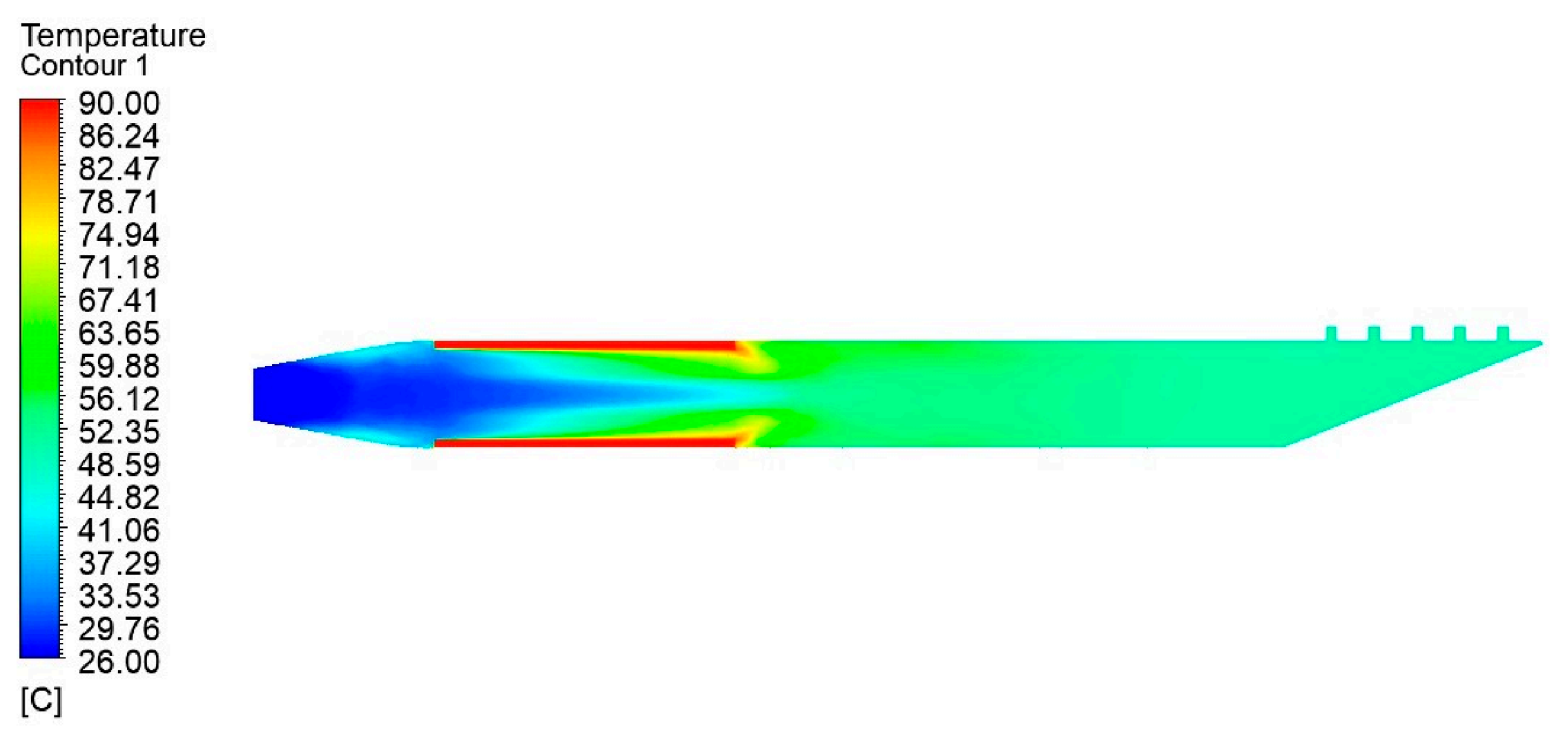
Shows the temperature of the air upon exiting the holes. The flow rate was set to 19 L/min, and the Peltier modules temperature was set to 90 °C.

**Figure 3:**
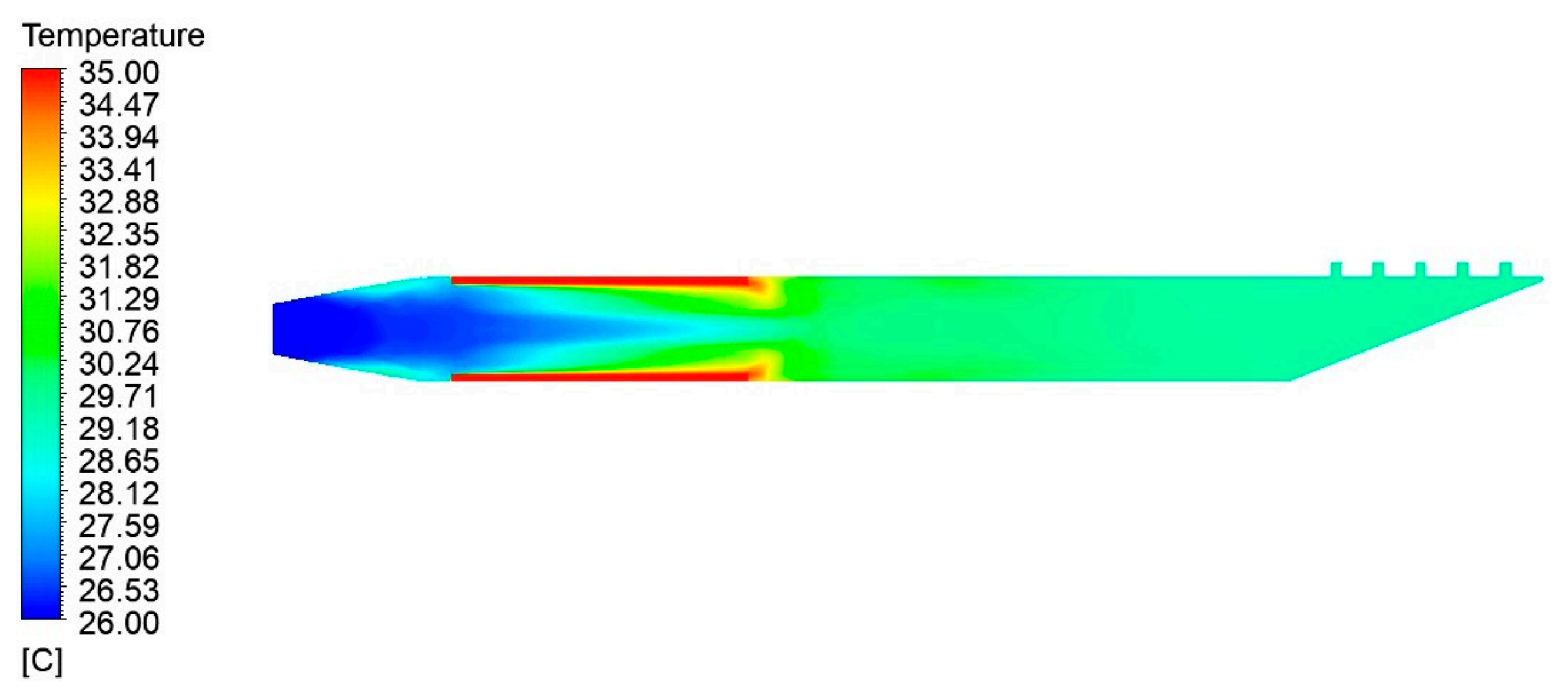
Shows the temperature of the air upon exiting the holes. The flow rate was set to 19 L/min, and the Peltier modules temperature was set to 35 °C.

**Figure 4:**
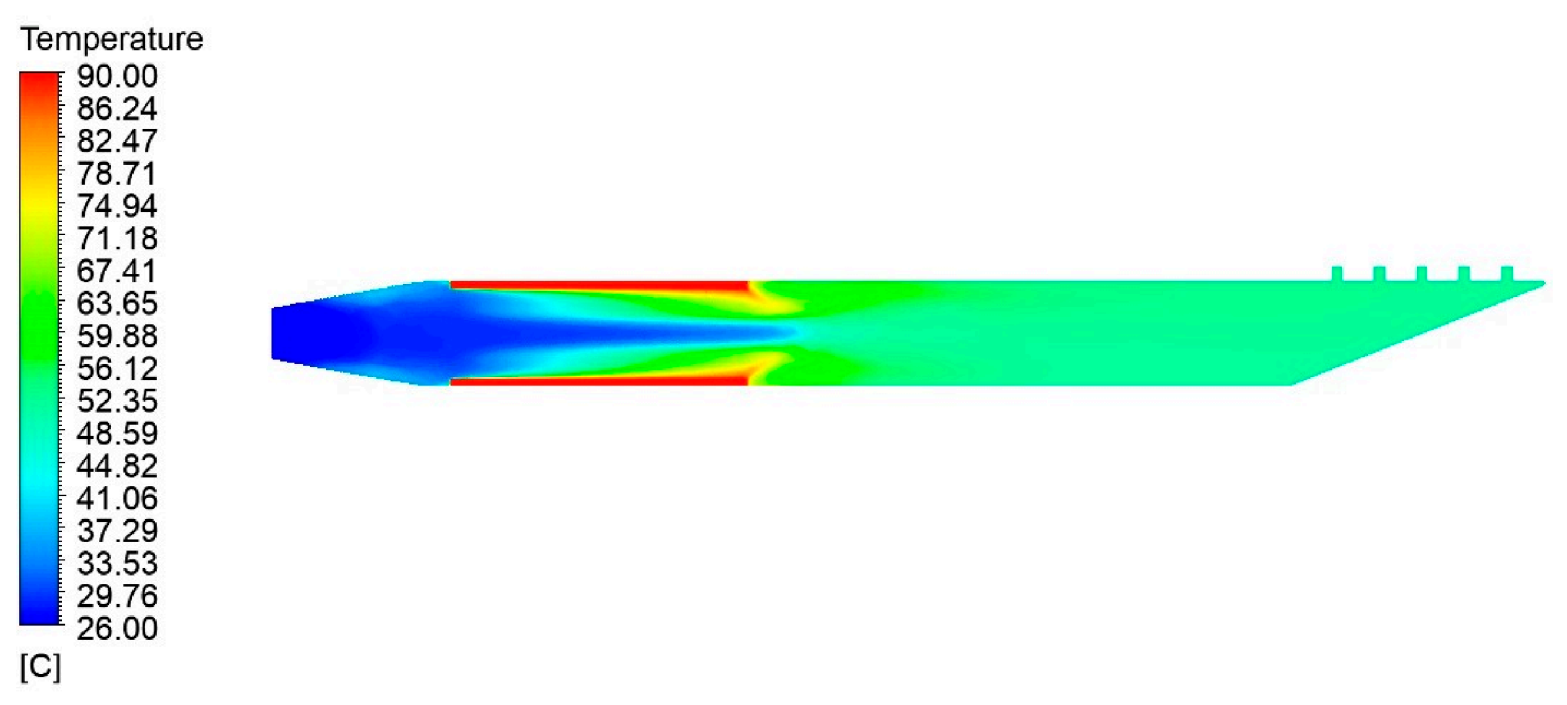
Temperature of the frequency-modulated air upon exiting the holes. The flow rate was set to 19 L/min, and the Peltier modules temperature was set to 90 °C.

Convergence was achieved when residuals dropped below 10^−6^ for continuity and momentum, and 10^−8^ for energy. This numerical configuration closely represents the actual operating conditions of the system and provides a reliable prediction of the coupled airflow and heat transfer behavior within the device. A sample output of the software for different states of the velocity, temperature, and frequency is demonstrated in Figure 2, Figure 3 and Figure 4.

### 2.2. Experimental Setup

As seen in Figure 1, the tactile surface is a 37.5 mm × 37.5 mm surface and features a total of 61 holes. Each hole has a diameter of approximately 1.5 mm, with a spacing of about 2.85 mm between each hole. The prototype was fabricated using 3D-printed ABS filament. Because ABS withstands temperatures up to approximately 100 °C, the operational temperature limit of the system was capped at 90 °C to ensure structural integrity and safe operation. This limit is enough to keep the material strong and ensure safe working conditions. Moreover, tests showed that 90 °C is enough for the project and experiments. The display is connected to the air source through two channels. In each channel, two TEC1-12705 thermoelectric devices (Peltier) are arranged in parallel, allowing air to flow between them, which causes the air to warm up and cool down [58,59,60]. The temperatures of the thermoelectric modules in the system are measured using an LM35 sensor, and this data can be quickly displayed via the interface. Additionally, the system uses two DC 22L 12V vacuum pumps. These vacuum pumps regulate the air pressure inside the device, allowing the user to experience the desired air-based tactile effect on their fingers. Furthermore, two 6V 150RPM Micro Gear DC Motors modulate the air pressure at the system’s input into a harmonic pressure, generating airflow at different frequencies. The system also includes two DC 555 12V micro vacuum pumps, used to create the olfactory effect. In addition, two normally closed mini solenoid valves were utilized to control the release of odor from the odor box.

**Figure 5:**
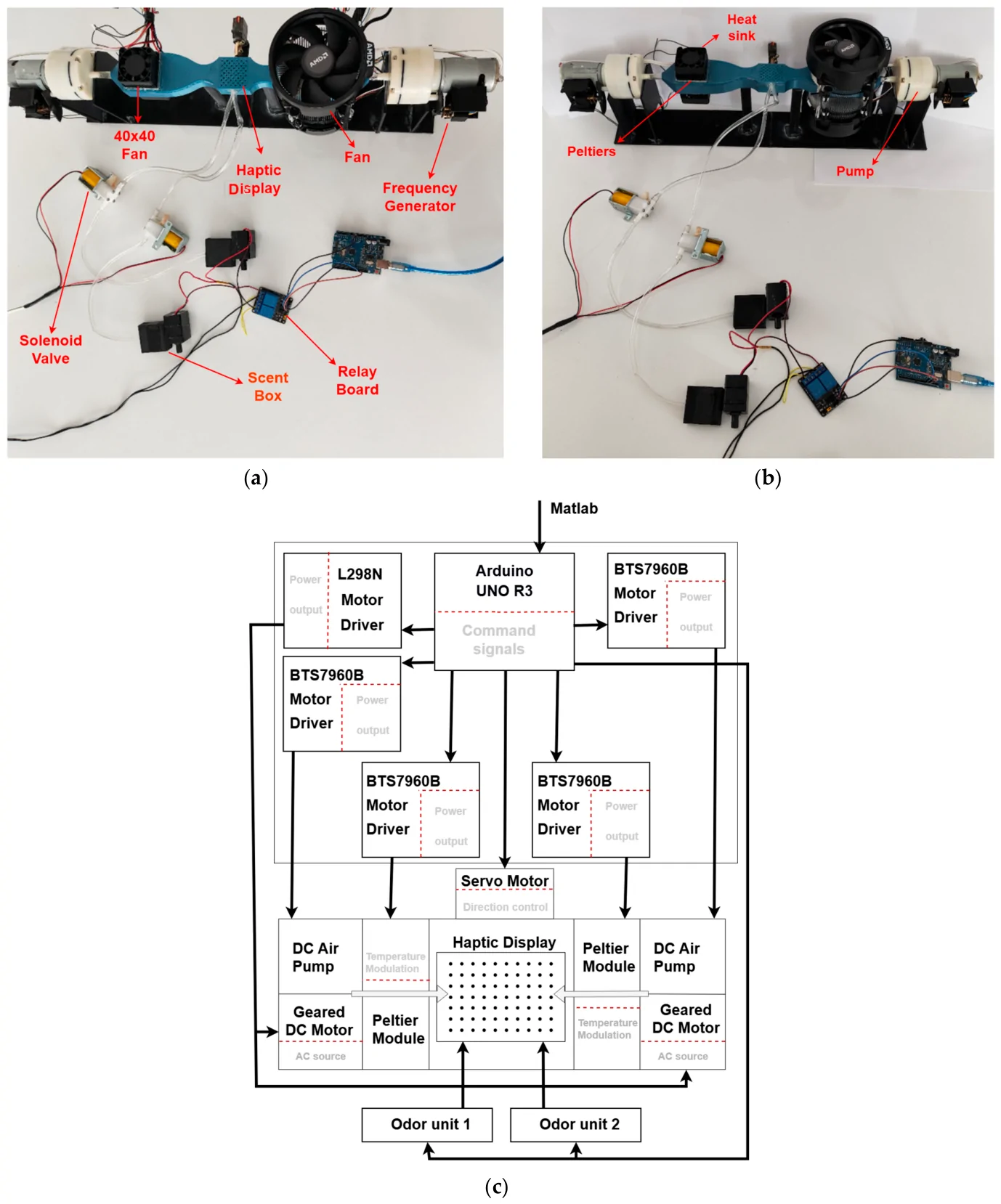
Shows developed tactile display and its components: (**a**) top view; (**b**) side view; (**c**) schematic representation of the developed system.

Another key component is the MG90S Servo. It is used to determine the direction of airflow in the bidirectional system. A 400-watt DC power adapter is used in the control unit to meet the energy requirements of all components in the system. The DC power adapter, together with four BTS7960B 40-amp motor driver modules, were specifically chosen to supply the high power needed for the Peltiers and air pumps. Additionally, an L298N motor driver board is used to control the two 6V 150RPM Micro Gear DC Motors. The motor was equipped with a half-circle panel that covers the intake nozzle input. By adjusting the speed of the motor, the desired frequency was achieved during the experimental evaluation. This design was chosen to mitigate the response latency caused by starting and stopping the motor directly. An Arduino Uno is used to control the system. Figure 5 shows developed device components and its schematic representation. A user interface was developed in MATLAB and Simulink R2023b to control all sensors and actuators in real time. The general specifications of the developed system are summarized in Table 1.

## 3. Experimental Evaluation

In the study, ten participants were invited to test the air-based tactile display system with olfactory features. Among the participants, six are male and four are female, with ages ranging from 18 to 35. All participants had no background in testing tactile displays. During the selection of participants, special attention was given to ensuring that individuals had no issues with their sense of smell or touch. The experiments were conducted based on the Declaration of Helsinki and approved by the Institutional Review Board of Istanbul Aydin University, and all participants provided informed consent prior to their participation.

### 3.1. Training

The training began with the introduction of the system to the users. During the introduction phase, the working principles of the tactile display system were explained to the users, and after providing information about the system components and the feedback mechanism, the sensory stimulus experience phase was initiated. Users were first exposed to airflows at different temperatures and asked to identify them. Then, the odors were presented, and they were encouraged to provide feedback on the odor perception process. They were also asked to perceive and identify frequency variations in the air-based stimuli.

### 3.2. User Study

**Figure 6:**
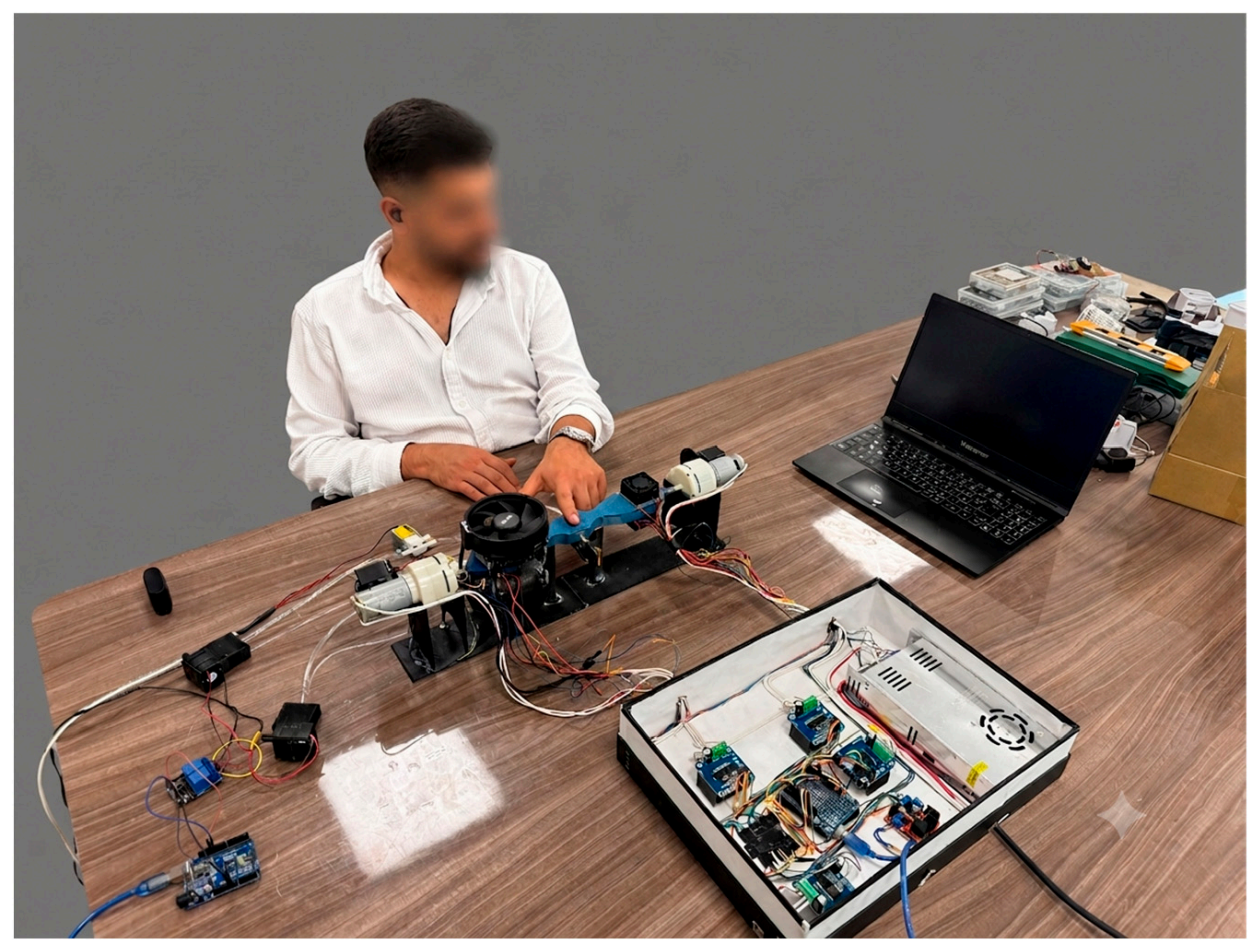
Subject testing the developed tactile display. The subject was asked whether the stimulus felt warm or cold with a pleasant or unpleasant odor.

A multi-stage testing process was conducted to evaluate the performance of the air-based tactile display with olfactory features developed in this study. As seen in Figure 6, participants sat comfortably in chairs. To prevent them from being affected by system sounds, each participant wore headphones and listened to white noise. Participants touched the display surface with their fingers and moved them across as if touching a surface like a smartphone screen. Although Figure 6 depicts a subject touching the surface with his left hand for illustration purposes, all participants in this study were right-handed and performed the tasks using their right hand. An interval of at least 30 s was maintained between consecutive trials while the experimental system was prepared for the next stimulus presentation. This interval also allowed any residual odor from the previous trial to dissipate before the subsequent presentation, thereby reducing the possibility of olfactory carry-over. All experiments were conducted in a naturally ventilated laboratory under consistent environmental conditions.

As the first stage of the test process, the lowest detectable DC airflow rate for each participant was determined at an ambient temperature of 26 °C. The ambient temperature was monitored and kept constant for all subjects during the experiment. Next, based on the identified minimum DC airflow rate, the system’s right side was activated to find the participant’s minimum perceivable cold temperature using the Adaptive Staircase method. Similarly, the system’s left side was activated to determine the minimum perceivable warm temperature. The purpose of this test was to assess each user’s thermal perception at the lowest detectable DC airflow.

In the next stage, a module (6V 150RPM Micro Gear DC Motors) capable of generating different frequencies was activated in the air supply input (AC airflow). With this addition, the airflow in the system became frequency-modulated. Using this new setup, each participant was first exposed to airflow at different frequencies while maintaining their previously determined minimum detectable DC airflow. The minimum frequency value perceived by each user was identified. Then, using the identified minimum frequency, the system’s different directions were activated to determine the participants’ minimum perceivable hot and cold temperatures. These tests were individually adjusted for ten participants, and a common airflow rate (19 L/min) and frequency (0.66 Hz) that all participants could perceive were determined.

For the established common frequency value (0.66 Hz), four different airflow rates (10 L/min, 13 L/min, 16 L/min, 19 L/min) were set, and the lowest perceivable hot and cold temperatures were determined. Additionally, in order to study the effect of the frequency on perceived temperature based on the determined common airflow rate (19 L/min), the lowest perceivable hot and cold temperatures were identified for participants at four different frequencies (0.66 Hz, 0.84 Hz, 1 Hz, 1.16 Hz) as well.

In the final stage of the experiment, each participant was randomly presented with thermal and olfactory stimuli under the DC airflow condition. Two distinct odors with clearly distinguishable smells were selected from commercially available products. Cotton pads soaked in each odor were placed inside two separate storage compartments integrated into the system’s olfactory module. Four stimulus combinations—warm–pleasant, cold–pleasant, warm–unpleasant, and cold–unpleasant—were created, and each combination was presented six times in a randomized sequence. During each experimental trial, participants reported their perceived thermal and olfactory sensations by selecting from a predefined set of written response options provided on a printed answer sheet. The use of standardized written response options ensured a consistent evaluation procedure across all participants and experimental conditions and facilitated reliable data collection. The collected feedback was used to assess the reliability and perceptual integration of thermal and olfactory cues within the developed airflow-based tactile display.

## 4. Results and Discussion

As a result of the first stage of the experiment, the mean minimum perceivable DC airflow rate was 12.48 L/min. The minimum perceivable DC cold air temperature and DC warm air temperature were determined to be 24.2 °C and 30.5 °C, respectively. The minimum frequency detected by the subjects was 0.66 Hz. Additionally, the minimum perceivable AC cold air temperature and AC warm air temperature were recorded as 17.03 °C and 33.2 °C, respectively. Frequency-modulated airflow caused participants to perceive warmth at higher and coldness at lower temperatures compared with continuous flow. This asymmetry can be attributed to the transient nature of convective heat transfer and the temporal sensitivity of cutaneous thermoreceptors, which could be a subject of interest for future research.

**Figure 7:**
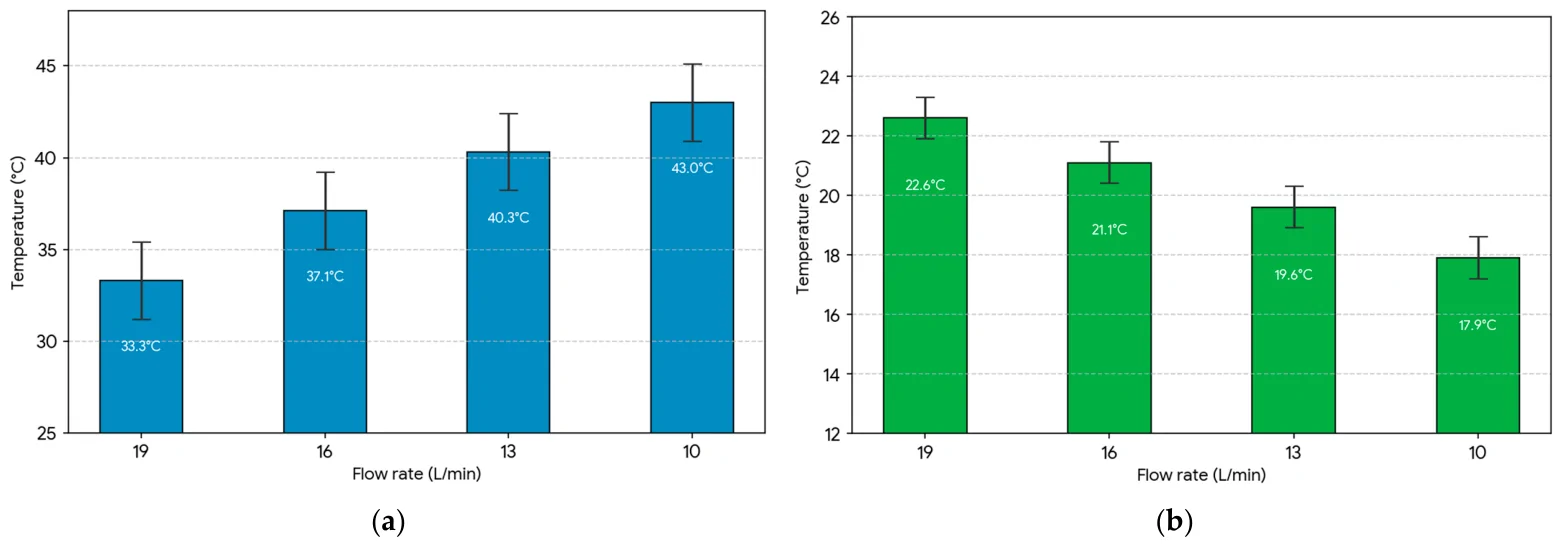
(**a**) Average perceived warm temperature for different flow rates; (**b**) average perceived cold temperature for different flow rates. Values are presented as mean ± standard deviation (SD) (n = 10).

The effects of a constant frequency (0.66 Hz) and different flow rates (10 L/min, 13 L/min, 16 L/min, 19 L/min) on warmness and coldness were analyzed. The warmness-flow graph in Figure 7a and the coldness-flow graph in Figure 7b show the temperature and coldness averages for four different flow rates. As seen in Figure 7a, as the flow rate decreases, there is a significant increase in temperature values. Additionally, a significant relationship is observed between different flow rate groups at a constant frequency (F[3,20] = 17.101, *p* = 0.001 < 0.05). Looking at each flow rate individually using post hoc analysis revealed no significant difference between the 10–13 L/min (*p* = 0.257 > 0.05), 19–16 L/min (*p* = 0.068 > 0.05), and 16–13 L/min (*p* = 0.149 > 0.05) groups. However, a significant difference is observed between the 19–13 L/min (*p* = 0.001 < 0.05), 19–10 L/min (*p* = 0.001 < 0.05), and 16–10 L/min (*p* = 0.003 < 0.05) groups. This demonstrates that a minimum flow rate difference of 6 L/min is required to distinguish variations in warmness.

When examining Figure 7b, a significant decrease in coldness values as the flow rate decreases was observed. Additionally, a significant relationship is observed between different flow rate groups at a constant frequency (F[3,20] = 34.459, *p* = 0.001 < 0.05). Further post hoc analysis at each flow rate individually yielded significant differences between groups, confirming that a flow rate of 3 L/min is required to distinguish variations in coldness.

The results obtained at a constant modulation frequency of 0.66 Hz revealed that increasing the airflow rate reduced the minimum perceivable warm temperature while increasing the minimum perceivable cold temperature. These findings suggest that airflow intensity modulates the perceived thermal strength primarily through its influence on the skin’s convective boundary layer and the rate of temperature change sensed by thermoreceptors [61,62,63].

**Figure 8:**
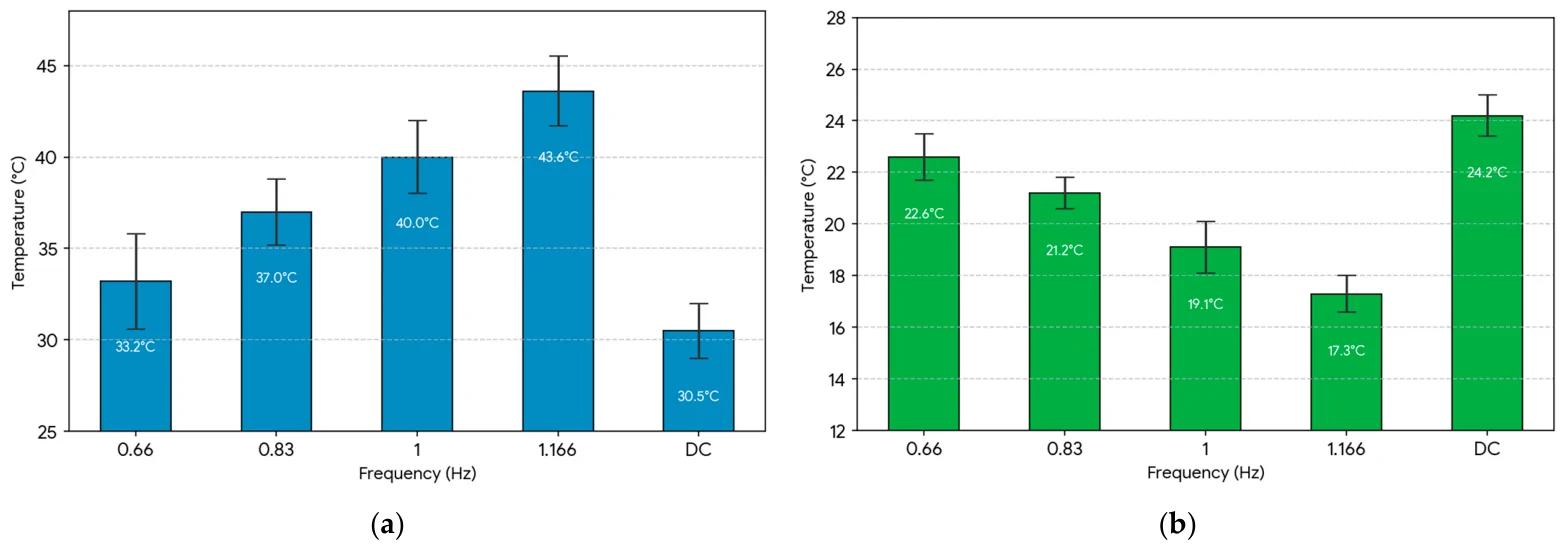
(**a**) Average perceived warm temperature for different frequencies and DC airflow; (**b**) average perceived cold temperature for different frequencies and DC airflow. Values are presented as mean ± standard deviation (SD) (n = 10).

Figure 8a,b illustrate the effects of constant flow rate (19 L/min) and different frequency values (0.66 Hz, 0.84 Hz, 1 Hz, 1.16 Hz) on warmness and coldness. In addition, the average DC perceived temperature is added to these figures for further comparison. In Figure 8a, a linear increase in temperature is observed with increasing frequency. Statistical analysis revealed a significant relationship between different frequency values (*p* = 0.001 < 0.05). When analyzing the groups individually, a significant difference is found between the 0.66–0.83 Hz (*p* = 0.020 < 0.05), 0.83–1 Hz (*p* = 0.048 < 0.05), and 1–1.166 Hz (*p* = 0.025 < 0.05) groups. Moreover, a significant difference is observed between the 0.66–1 Hz (*p* = 0.001 < 0.05), 0.66–1.166 Hz (*p* = 0.001 < 0.05), and 0.83–1.166 Hz (*p* = 0.001 < 0.05) groups. The findings show that a 0.17 Hz variation in frequency is sufficient to distinguish warmness variation.

In Figure 8b, a significant decrease in coldness is observed as the frequency of the incoming fluid increases, indicating that a lower temperature is required when the frequency of flow increases. It also shows that frequency is significant for perceiving coldness at a constant flow rate (F[3,20] = 53.441, *p* = 0.001 < 0.05). Further post hoc analysis yielded the same results, confirming that a frequency modulation of 0.17 Hz is required to distinguish variations in coldness.

When the flow rate is held constant at 19 L/min, increasing the modulation frequency from 0.66 Hz to 1.16 Hz produced an opposing shift in warm and cold detection thresholds: cold thresholds decreased (22.6 to 17.3 °C), while warm thresholds increased (33.2 to 43.6 °C). This behavior is consistent with unsteady convective heat transfer and the finite temporal integration of cutaneous thermoreceptors. These results indicate that, at a given flow magnitude, the temporal structure of airflow is a key determinant of perceived thermal quality and must be considered when designing airflow-based haptic stimuli [64,65,66,67,68].

As seen in Figure 8a for warmness, higher temperatures could be perceived with frequency-modulated airflow than with DC. In addition, in Figure 8b for coldness, lower temperatures could be perceived with frequency-modulated airflow than with DC. A repeated-measures ANOVA test was performed to check if there is a significant difference between AC and DC airflow considering temperature. The results revealed that for both conditions, there was a significant difference between the two stimuli.

Finally, a test phase was created to assess the random effects on users in the DC condition. Participants correctly identified 21.5 ± 1.7 out of 24 thermal–olfactory combinations, corresponding to a mean accuracy of approximately 90% (±7%). This indicates that most users were able to reliably distinguish the paired stimuli presented by the airflow-based display.

## 5. Conclusions

This study presented the design, implementation, and initial perceptual characterization of an airflow-based multimodal thermal–tactile–olfactory display under controlled laboratory conditions. By evaluating the system under continuous (DC) and frequency-modulated (AC) airflow conditions, we identified key parameters governing thermal perception. Specifically, at a constant modulation frequency of 0.66 Hz, higher airflow rates decreased the minimum perceivable warm temperature while increasing the minimum perceivable cold temperature, demonstrating enhanced convective heat transfer. Conversely, under fixed airflow rates, increasing modulation frequency raised warm thresholds and lowered cold thresholds, highlighting the roles of transient heat transfer and thermoreceptor temporal sensitivity. Although these physical trends are clear, dedicated psychophysical studies are still required to fully isolate the underlying sensory mechanisms.

As a technical proof-of-concept, the prototype establishes the feasibility of using airflow as a unified medium for tactile, thermal, and olfactory feedback. However, this integrated approach introduces inherent trade-offs, as airflow conditions that optimize tactile cues may not align with those optimal for thermal or scent delivery. Although the selected airflow range of 10–19 L/min provided an effective operating balance for baseline characterization, further work is required to systematically quantify these cross-modal interactions and define parameters for complex haptic tasks, such as texture rendering.

The current prototype also presents several technical limitations that define its scope as an early-stage benchtop platform. The thermoelectric modules contribute to overall system bulk and a relatively slow thermal response time, limiting immediate portability and rapid stimulus transitions. The prototype exhibited a thermal response time of approximately 15 s for heating and 20 s for cooling, which may limit rapid thermal transitions in applications requiring fast feedback. Furthermore, the airflow control and odor release mechanisms require further optimization for precision and safety. Future technical iterations will focus on hardware miniaturization, closed-loop thermal control, and refined olfactory delivery. Incorporating real-time skin temperature monitoring, thermal imaging, and additional vibrotactile dimensions will provide deeper insight into the thermal–tactile coupling at the skin interface.

Compared with recent airflow-based haptic systems—such as TWIRL [50] and FIRE [53], which focus primarily on hardware miniaturization or application-specific demonstrations—this work contributes a systematic perceptual evaluation of airflow rate and modulation frequency. The perceptual thresholds identified here provide foundational design guidelines applicable to future airflow-based thermal displays, regardless of the underlying actuation technology.

Crucially, this study was designed strictly as a fundamental perceptual characterization rather than an application-specific validation. Downstream applications—such as virtual reality (VR), augmented reality (AR), telepresence, and human–robot interaction—have not yet been empirically tested with this system. Future studies must evaluate user experience, task performance, and sense of immersion within these interactive environments to validate the practical utility of the proposed display. Overall, this work serves as a proof-of-concept for multimodal airflow-based rendering, establishing baseline perceptual guidelines upon which future compact, contact, or noncontact interfaces can build.

## Figures and Tables

**Table 1:** General specifications of the developed device.

Technical Parameter	Specification
Overall dimensions (W × L × H)	155 × 600 × 175 mm
Weight	1.4 kg
Power consumption during operation	170 W
Cooling response time (25 → 18 °C)	≈20 s
Heating response time (25 → 43 °C)	≈15 s

## Data Availability

The datasets presented in this article are not readily available due to technical/time limitations.
